# Carbon metabolism and niche adaptation in *Streptococcus pyogenes* pathogenesis

**DOI:** 10.1128/msphere.00668-25

**Published:** 2026-03-31

**Authors:** Wei Xu, Cheryl Y. M. Okumura

**Affiliations:** 1 Department of Biomedical Sciences, Marshall University Joan C. Edwards School of Medicine 12354, Huntington, West Virginia, USA; 2 Biology Department, Occidental College 5121, Los Angeles, California, USA; University of Minnesota Twin Cities, Minneapolis, Minnesota, USA

**Keywords:** *Streptococcus pyogenes*, carbon metabolism, pathogenesis, virulence, immune evasion, niche-specific, translational

## Abstract

Responsible for over 500,000 deaths annually around the world, *Streptococcus pyogenes* (group A *Streptococcus* [GAS]) infections have resurged in the post-COVID-19 era due to immune debt and the rise of strains with enhanced adaptive capabilities. The formidable pathogenicity of GAS is fueled by metabolic plasticity that coordinates virulence with niche-specific adaptation. In this minireview, we dissect how GAS functions as a sophisticated metabolic decision-maker, revealing survival strategies of the bacteria that allow persistence and vulnerabilities that can be targeted for therapeutic development. From the oropharynx to the bloodstream, niche-specific carbon sources and availability dictate downstream biosynthetic processes, creating an integrated metabolic network that controls pathogen fitness. Dynamic shifts in central carbon metabolism are orchestrated by an expanded repertoire of global regulators that directly couple nutrient availability to virulence factor expression. The resulting bacterial metabolic byproducts serve as dual-purpose weapons, limiting competition with commensal microbes and reprogramming host cell immune responses. The ability of GAS to fine-tune and couple metabolism to niche-specific survival factors reveals pathogen-specific targets that can be exploited for therapy. We evaluate high-potential therapeutic strategies that aim to disrupt this critical metabolism-virulence nexus. The development of these precision anti-virulence strategies to counter GAS infections is critical in an era of rising antimicrobial resistance.

## INTRODUCTION

The Gram-positive bacterial pathogen *Streptococcus pyogenes* (group A *Streptococcus* [GAS]) imposes a dual burden on global health through both acute infections and post-infectious sequelae (1). Its clinical spectrum spans from superficial diseases such as pharyngitis and impetigo to severe invasive infections, including necrotizing fasciitis and streptococcal toxic shock syndrome (STSS). Post-streptococcal complications such as rheumatic heart disease (RHD) and acute glomerulonephritis contribute to the burden of GAS diseases (2). Annually, GAS accounts for over 500,000 deaths worldwide, with RHD responsible for 319,400 deaths in resource-limited regions where delayed diagnosis and limited healthcare access exacerbate outcomes (3). Alarmingly, in the post-COVID-19 era (2022–2024), hospitals and clinics have witnessed a global resurgence of invasive GAS infections, exemplified by a sharp increase in pediatric cases in the UK (4) and a rise in STSS mortality (5, 6). This surge, attributed to both population-wide “immune debt” from reduced pathogen exposure during lockdowns and the emergence of successful epidemic lineages (7), underscores the urgent need to re-examine GAS pathogenesis. As a human-specific bacterium that has evolved with and adapted to its host environment, it is critical to examine GAS pathogenesis through the lens of its most potent survival and virulence-regulating trait: carbon utilization.

**Fig 1:**
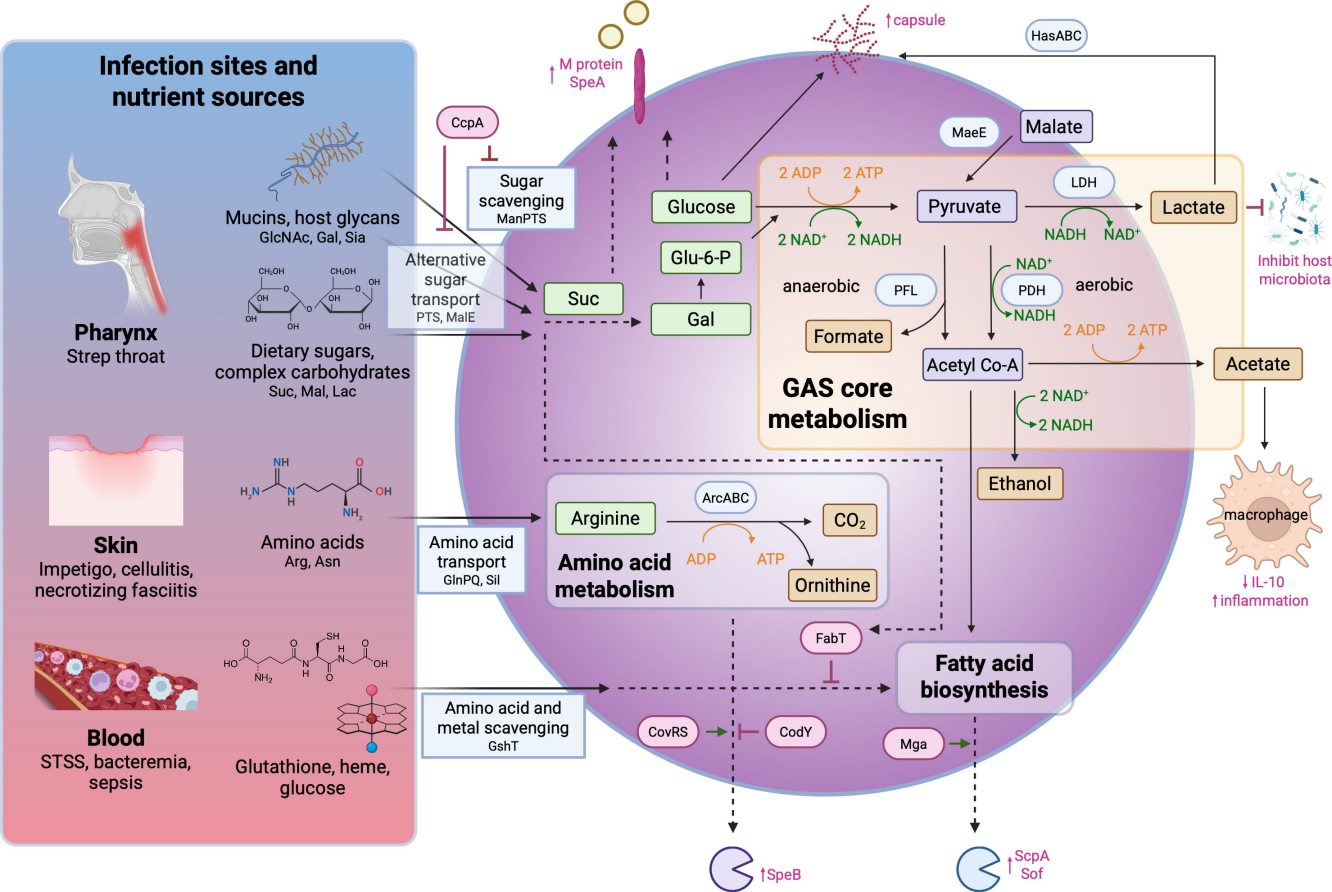
The central metabolic decision-making network of GAS links carbon source availability to virulence expression. Carbon sources depicted include sucrose (Suc), maltose (Mal), lactose (Lac), N-acetylglucosamine (GlcNAc), galactose (Gal), and sialic acid (Sia). Environmental and nutrient cues shape carbon flux through core pathways, including glycolysis, homolactic fermentation (LDH), and mixed-acid fermentation (PDH/PFL), which together define GAS core metabolism. Under nutrient-limited conditions, metabolic flux is reprogrammed to promote amino acid catabolism (e.g., the ADI pathway) and to downregulate fatty acid biosynthesis. Metabolic intermediates and end-products function as signaling molecules that modulate the activity of global regulators (indicated in magenta), thereby coupling metabolic state to transcriptional control. These regulators directly govern the expression of key virulence determinants, including the hyaluronic acid capsule, exotoxins (SpeA, SpeB), and other immunomodulatory effectors, coordinating virulence programs with metabolic capacity and environmental adaptation. Dotted arrows indicate pathways not depicted in detail. Created with BioRender.com.

GAS thrives in diverse host niches, from carbohydrate-rich mucosa, to nutrient-restricted dermis, to nutrient-poor conditions in the bloodstream, by dynamically reprogramming central carbon metabolic pathways (8) and adapting its carbon metabolism to local nutrient availability (Fig. 1). Rapid shifts between homolactic fermentation and mixed-acid fermentation (9), along with efficient exploitation of host-derived carbon sources such as mucosal glycans (10, 11) allow bacteria to harvest and utilize a variety of carbon sources. The flux through these core pathways is additionally influenced by the availability of other nutrients such as amino acids, which can serve as alternative carbon substrates or regulatory signals (Fig. 1).

Metabolic plasticity is governed by streamlined pathways for carbohydrate fermentation and scavenging under precise regulatory control that directly coordinates carbon source selection with virulence factor expression (12). Beyond established regulators such as carbon catabolite protein A (CcpA) and control of virulence sensor/regulator (CovRS) (13), recent studies reveal that carbohydrate availability directly modulates virulence (14) through regulators such as regulator of F (RofA) (15) and post-translational modification of multiple gene activator (Mga) by the phosphotransferase system (PTS) (16), first demonstrated in M1 and M4 serotypes and confirmed in M59 GAS (17, 18). This coordination ensures that virulence is deployed as a direct function of carbon metabolic capacity, making it a central processing unit for pathogenic decision-making. Furthermore, the output of carbon metabolism, such as precursor availability for lipid synthesis, directly impacts the production of virulence factors and other bacterial processes critical for survival and immune evasion (19).

The direct link between carbon metabolism and virulence in GAS exposes a fundamental vulnerability. The pathogen’s dependence on specific nutrient scavenging pathways and central carbon flux presents opportunities for novel therapeutic strategies to disrupt its energy generation, virulence regulation, and niche adaptation. This review synthesizes advances from the past 5 years in our understanding of how GAS metabolic decision-making translates to disease outcomes, revealing novel targets for treating infection. We will examine (i) niche-specific programs that enable metabolic plasticity across host environments, (ii) the signaling role of key metabolic byproducts in the regulation of virulence and host immunity, and (iii) evaluate the promise and challenges of therapeutic strategies that target this core metabolic-virulence axis. Further investigation into these key areas will catalyze the development of precision therapies that exploit GAS’s metabolic vulnerabilities while mitigating antibiotic resistance.

## NICHE-SPECIFIC CARBON SOURCES AND METABOLIC PROGRAMS

GAS exhibits remarkable metabolic plasticity, tailoring its core fermentative pathways and substrate preferences to the distinct nutritional landscapes of its primary host niches (20).

### Core fermentative pathways for energy generation

Under nutrient-rich and culture conditions, GAS relies predominantly on glycolysis (Embden-Meyerhof-Parnas pathway) for energy production, generating ATP and pyruvate. The absence of a complete tricarboxylic acid (TCA) cycle in this obligate fermenter necessitates pyruvate diversion into fermentation to regenerate NAD^+^ (21). Under anaerobic conditions, lactate dehydrogenase (LDH) converts pyruvate to lactate, acidifying the microenvironment and potentially suppressing competing microbiota (Fig. 1). However, GAS dynamically shifts its metabolism in response to oxygen availability, redox status, and carbohydrate limitation. Regulators such as Rex (22) sense the intracellular NADH/NAD^+^ ratio to coordinate gene expression (23). Under microaerophilic or oxygenated conditions, pyruvate formate lyase (PFL) activity decreases, while pyruvate dehydrogenase (PDH) becomes active, oxidizing pyruvate to acetyl-CoA (Fig. 1). Acetyl-CoA is then converted to acetate via phosphotransacetylase and acetate kinase to yield ATP, or to ethanol via alcohol dehydrogenase, regenerating NAD^+^. This flexibility optimizes ATP yield and redox balance in response to environmental change.

Metabolic cues directly control the expression and activity of GAS’s primary virulence arsenal, ensuring energy resources are allocated to pathogenicity only when advantageous. GAS infection outcomes are, therefore, dictated by the pathogen’s ability to tailor its carbon metabolism to the specific nutritional landscape of each host niche. The oropharynx, skin, and bloodstream present unique challenges and resources (Fig. 1). GAS not only survives but also thrives by executing precise, niche-specific metabolic programs, and core fermentative pathways subsequently provide the metabolic flexibility to drive the appropriate virulence response for each environment.

### Oropharyngeal colonization: scavenging mucosal glycans to mediate adhesion

The human oropharynx is colonized by a diverse array of microbial species, creating a competitive environment for space and nutrients, as well as the hostile production of antimicrobial toxins and compounds by commensal microbes (24, 25). Virulence factor expression for adhesion and immune evasion is, therefore, critical for survival but is metabolically expensive processes. Free glucose is extremely limited in this niche; in healthy individuals, fasting salivary glucose is typically <1 mg/dL (26), which is over 100-fold lower than blood glucose levels. Consequently, the primary abundant carbon sources in the oropharynx are glycans—complex carbohydrate structures on host cell surfaces and in mucosal secretions. GAS efficiently scavenges these host glycans, including those from mucins, for colonization (16).

GAS recognition and binding to host glycan patterns, such as ABO(H) and Lewis antigens on oral epithelial cells (27), means successful colonization is directly dependent on the ability to exploit local carbon sources. GAS can utilize alternative sugars such as lactose, sucrose, and maltose via phosphoenolpyruvate PTS (28), ensuring energy production continues when glucose is absent. GAS can also utilize dietary sugars via systems such as the maltose transporter MalE; *malE* mutants show colonization defects, confirming the dependence on scavenging these complex carbohydrates in the oropharynx (29). The potential impact of dietary changes on bacterial metabolic adaptation is, therefore, an area for future investigation. Additionally, GAS repurposes transporters such as the mannose phosphotransferase system (Man-PTS) to acquire mucin-derived sugars, including N-acetylglucosamine (GlcNAc), galactose, and sialic acid (11, 16) (Fig. 1).

This glycan scavenging is strategic for virulence factor expression. Galactose is funneled through the Leloir pathway, conserving free glucose for the synthesis of the hyaluronic acid capsule (30), a critical investment in immune evasion through molecular mimicry (Fig. 1). The expression of the hyaluronic acid capsule via the *has* operon is further promoted by acidification of the local environment by lactate accumulation from fermentation (31) (Fig. 1). A key surface virulence factor whose expression is tightly linked to carbohydrate metabolism is M protein, a fibrillar adhesin essential for immune evasion and epithelial attachment. The import of PTS-transported sugars such as glucose and sucrose stimulates the synthesis of M protein (32) and is linked to glycolytic flux (Fig. 1). Robust glycolytic flux generates the ATP required for the stable anchoring of M protein to host receptors (33, 34).

### Skin and soft tissue infections: catabolizing host proteins

The dermal environment presents a distinct metabolic challenge. While interstitial fluid glucose concentrations are similar to those in blood (~5.5 mM or 100 mg/dL in a normoglycemic state) (35), access to this carbon source is limited by tissue architecture and host defenses. Consequently, this niche is effectively glucose-restricted for colonizing bacteria, but rich in alternative nutrients such as proteins and amino acids (36, 37). Here, GAS pivots from glycolysis to amino acid catabolism as its primary carbon and energy source (38) (Fig. 1). Transcriptomic data from murine necrotizing fasciitis models reveal a profound metabolic shift: a significant upregulation of transporters for alternative sugars (39) and a nearly sixfold induction of the arginine deiminase (ADI) pathway (40).

The ADI pathway is a major contributor to skin pathogenesis. By catabolizing host-derived arginine sourced from filaggrin breakdown, GAS simultaneously generates ATP, neutralizes acid stress, and most crucially, upregulates exotoxin genes like *speB* (40) (Fig. 1). The cysteine protease SpeB is essential for bacterial survival in skin and soft tissues and causes gasdermin A-dependent pyroptosis in keratinocytes (41). Thus, this direct coupling of arginine as a carbon source to skin cell cytotoxicity is a primary driver of tissue destruction.

The availability of specific amino acids can also profoundly influence carbon metabolism and virulence. Tissue invasion triggers host endoplasmic reticulum (ER) stress, elevating extracellular asparagine levels (42). Asparagine availability is sensed by GAS via the GlnPQ transporter and Sil system, leading to CovRS-dependent upregulation of virulence genes, effectively linking amino acid sufficiency to the carbon metabolism-driven virulence program (43) (Fig. 1). Inhibiting this host stress response reduces bacterial burden, proving GAS co-opts host metabolism to fuel its invasion (42). Similarly, under branched-chain amino acid replete conditions, CodY can bind DNA to repress the expression of virulence genes such as SpeB and those encoding extracellular nucleases, but this repression can be relieved upon limitation of such amino acids (44) (Fig. 1). Thus, the ability to sense and utilize alternative carbon sources such as amino acids is directly linked with the expression of bacterial genes crucial for survival in these environments.

### Systemic dissemination: survival in the bloodstream

Despite homeostatic blood glucose levels of ~5.5 mM (100 mg/dL), the bloodstream presents a nutrient-scarce and immune-dominated hostile environment for GAS (45), as free sugars and other essential nutrients are compartmentalized and not readily accessible to invading bacteria. Immune evasion strategies are metabolically costly. Production of the secreted endoglycosidase EndoS, which cleaves IgG N-glycans to impair opsonization (46), represents a significant investment of cellular resources. Resources are also allocated to activities like streptolysin S (SLS)-mediated platelet activation (47), which contributes to the pathology of streptococcal toxic shock syndrome (STSS) (45). Survival in the blood, therefore, requires metabolic resilience focused on the efficient scavenging of essential nutrients (48), while maintaining redox balance and virulence factor expression (Fig. 1).

In the bloodstream, GAS relies on salvaging host glutathione (GSH) (49) via the GshT transporter. Glutathione not only serves as a powerful antioxidant to combat oxidative stress but also influences central carbon metabolism. Impaired glutathione uptake forces GAS to rewire its metabolic pathways, increasing NADPH production and altering the expression of key virulence factors (49).

Beyond carbon, systemic dissemination also depends on acquiring metals as enzymatic cofactors. Since free iron is not available, the Shr protein steals heme from hemoglobin to satisfy the intense iron demand in this metal-restricted environment (50, 51). Adaptations in lipid metabolism, such as those controlled by the FabT regulator, are also crucial for survival in the blood (19). FabT controls fatty acid biosynthesis, which, in turn, limits the levels of incorporation of toxic host-derived saturated fatty acids into bacterial membranes and is linked with the expression of various Mga-controlled virulence factors required for persistence (19) (Fig. 1). These nutrient acquisition systems illustrate the breadth of metabolic adaptation required for systemic dissemination.

## METABOLIC RE-PROGRAMMING OF GAS AND THE HOST RESPONSE

The pathogenic success of GAS is determined by its ability to coordinate virulence factor expression with its metabolic state. Carbon flux through core pathways generates specific metabolic intermediates that function as direct regulators of virulence (52, 53). The production of fermentation byproducts also coordinates the expression of key virulence determinants and manipulates the host immune response.

### Regulatory networks linking carbon availability to virulence

Carbon metabolic pathways are coordinated with virulence through regulators that sense both nutrient and environmental status (Fig. 1). The CcpA is the master regulator of carbon catabolite repression (54). In glucose-replete conditions, CcpA binds cre sites to repress genes in the *mae* operon and the ADI pathway (54) (Fig. 1). This prioritizes glycolytic flux and represses scavenging pathways for alternative carbon sources until needed. Environmental cues can also direct bacterial metabolic choice. For example, GAS core carbon metabolism can acidify the microenvironment; additionally, niches such as the skin or vaginal mucosa are naturally acidic. Under acidic stress, the MaeKR two-component system activates the *mae* operon, where the enzyme MaeE converts malate to pyruvate, providing carbon and energy (55) (Fig. 1). Deletion of *maeE* enhances murine virulence, indicating a trade-off between acid resistance and immune evasion.

The regulation of exotoxins is another demonstration of the linkage between nutrient availability and virulence. CcpA suppresses SpeA expression (56) in glucose-rich environments but relieves this repression during carbohydrate limitation (10) (Fig. 1). This ensures costly toxin production is reserved for states of metabolic stress, aligning hyperinflammation with the need to liberate nutrients from host tissues. SpeB (57, 58) is another key example of carbon-dependent regulation; repressed by CcpA under high glucose, SpeB is activated by the dual signals of glucose depletion and lactate accumulation. This activation enables GAS to degrade host proteins, neutralizing defenses and liberating amino acids and carbon sources for catabolism.

In nutrient-limited host environments, GAS activates pathways to exploit non-glycolytic carbon sources (40, 59), a switch often directly linked to virulence. In addition to relief of CcpA repression of scavenging pathways, the PTS extends nutrient sensing beyond glucose (60). Phosphorylation of PRD-containing regulators (PCVRs) like RofA (15) in low glucose conditions downregulates pilus genes and upregulates capsule biosynthesis, a strategic shift from adhesion to immune evasion dictated by carbon availability. Similarly, genetic evidence suggests the global virulence regulator Mga is likely phosphorylated by the mannose PTS, linking its activity to carbohydrate availability and thereby coupling quorum-sensing with virulence gene expression (61).

Multi-omic analyses, including the identification of independently modulated gene sets (iModulons) (62), demonstrate that shifts in carbon sources activate regulatory modules associated with the CovRS system (63). Furthermore, genome-scale metabolic models (GEMs) are now providing systems-level insights into these regulatory networks, predicting essential metabolic functions and niche-specific adaptations (64). These studies provide genome-scale evidence that carbon metabolic state is a primary input for the virulence gene regulatory network.

### Metabolic effectors of the host environment and response

The foundational metabolic repertoire of GAS not only provides energy but also generates specific intermediates that act as direct regulators of virulence and modulators of disease pathogenesis (Fig. 1). Lactate accumulation, for example, creates an acidic niche that suppresses the growth of competing flora and promotes GAS capsule synthesis (65). However, these metabolic byproducts can also signal to host cells, profoundly affecting their action and overall disease outcome.

For the host, lactate can function as an immunological signal. Recent work indicates that lactate from *Staphylococcus aureus*, a conserved danger signal, can be shuttled into neutrophil mitochondria, promoting reactive oxygen species (ROS) production and the formation of neutrophil extracellular traps (NETosis) (66). However, the specific impact of GAS-derived lactate on processes such as neutrophil chemotaxis requires further investigation, as its immunomodulatory role may be context-dependent and distinct from that of other metabolites (65, 67, 68). Within macrophages, lactate can also induce histone lactylation, an epigenetic modification that may reprogram inflammatory cytokine production (69). However, the *in vivo* significance of this modification in GAS infection remains an open question.

Acetate, generated from acetyl-CoA, serves as a critical immunomodulatory metabolite (50). Data from GAS infection models demonstrate that acetate and formate production suppress the production of host anti-inflammatory cytokine IL-10, shifting the immune response toward a pro-inflammatory state that may facilitate tissue damage and nutrient release (67). Strains with defective acetate production elicit increased IL-10 responses, a phenotype reversible by acetate supplementation. This metabolic crosstalk is evidenced using histone deacetylase (HDAC) inhibitors, which mimic acetate’s effects to abolish IL-10 production in infected macrophages (67). This immunomodulation occurs not through classic histone acetylation, but likely via acetate altering host acetyl-CoA pools and modulating the activity of metabolic enzymes, representing a form of non-genetic host metabolic reprogramming. Thus, GAS carbon metabolism can rewire the host’s epigenetic landscape to favor a pro-inflammatory state. The interplay between lactate and acetate, a short-chain fatty acid (SCFA), also exemplifies a higher-order strategy of balance that allows GAS to fine-tune the inflammatory response to its benefit. This strategy may also be important in other environments where SCFAs from commensal species are abundant.

## THERAPEUTIC STRATEGIES TARGETING GAS CARBON METABOLISM

**Fig 2:**
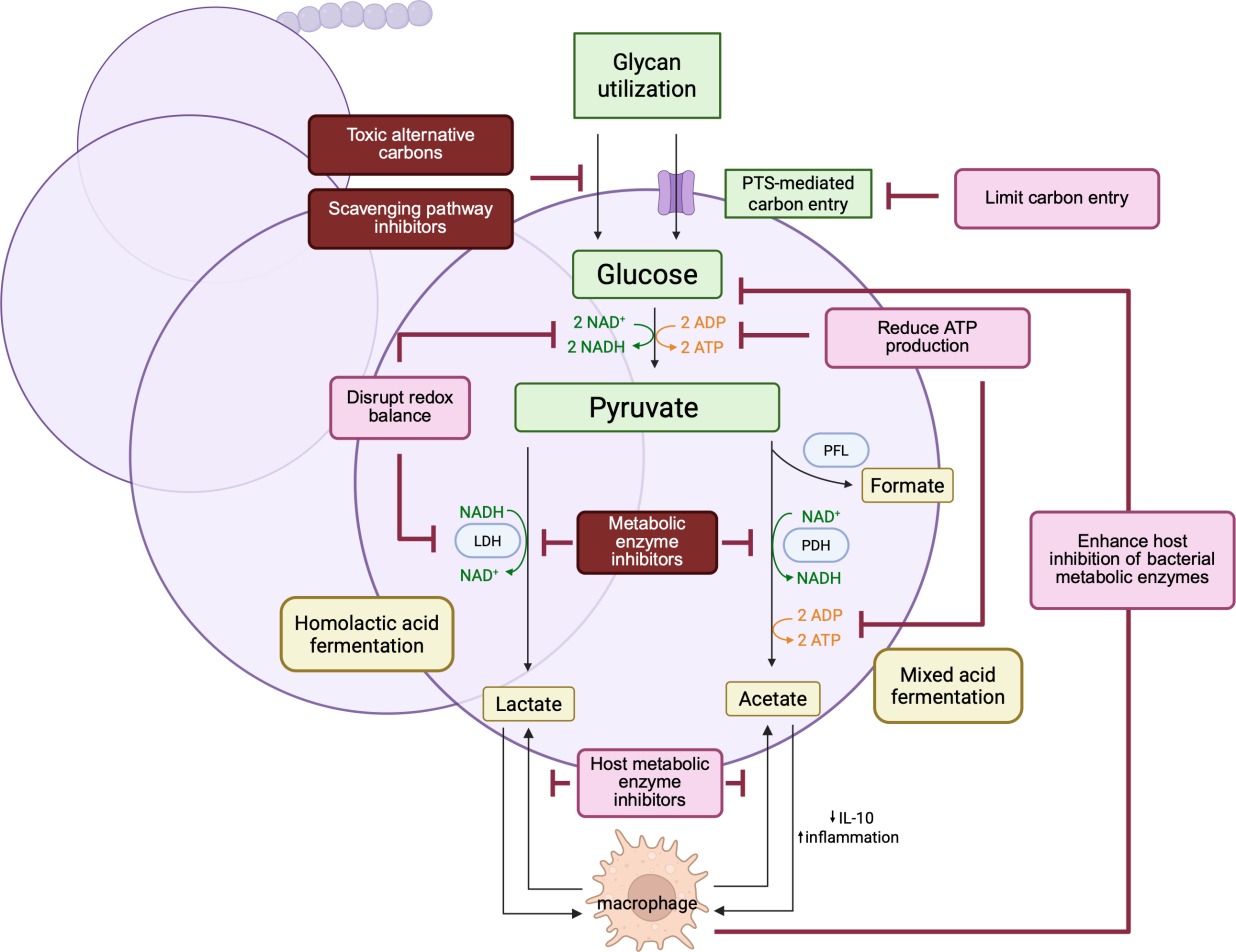
Therapeutic strategies targeting metabolic vulnerabilities of GAS. The essential link between metabolism and virulence reveals actionable targets for novel anti-infective approaches. Strategic bacterial targets (dark red boxes) include scavenging pathway and metabolic enzymes. Other strategies (pink boxes), including disrupting bacterial redox balance and ATP production, or targeting host processes such as limiting host lactose generation or enhancing the production of bacterial metabolic inhibitors, may suppress virulence while reducing selective pressure for drug resistance. Created in BioRender.com.

The direct link between carbon metabolism and virulence in GAS exposes a fundamental vulnerability. The pathogen’s dependence on specific nutrient scavenging pathways and central carbon flux presents opportunities for novel therapeutic strategies to directly disrupt bacterial metabolism or alter host responses to bacterial metabolic products (Fig. 2).

### Targeting bacterial metabolism

Direct targeting of core metabolic enzymes offers a path to disrupt energy production and virulence (Fig. 2). Key enzymes in central carbon metabolism—including LDH, PFL, and PDH—represent attractive targets whose inhibition could simultaneously impair ATP generation, disrupt redox balance, and reduce bacterial fitness across multiple host niches. The essential NADPH-generating enzyme GapN (70) is a validated target. Antisense peptide nucleic acids (asPNAs) against *gapN* reduce bacterial counts by >90% *in vitro*, including in macrolide-resistant strains (70). In nutrient-scarce environments, targeting scavenging pathways could reduce bacterial fitness. The ADI pathway**,** critical for skin pathogenesis, is an example of a high-value target (71): inhibition would simultaneously impair ATP generation, acid tolerance, and SpeB production. Furthermore, the ADI pathway contributes to antibiotic tolerance in biofilms (72); its inhibition could, therefore, sensitize GAS to conventional antibiotics, providing a synergistic therapeutic strategy. Even common compounds can disrupt carbon metabolism. Polyols such as xylitol inhibit growth of clinical GAS isolates from peritonsillar abscesses in a concentration-dependent manner, suggesting potential for prophylaxis through dietary modification (73) (Fig. 2).

### Disrupting metabolic signals that modulate host responses

Strategies to interrupt the signaling function of carbon metabolites can disarm the pathogen without being bactericidal, preventing the development of drug resistance. Targeting the immunomodulatory role of acetate is one such example. Inhibition of bacterial PDH reduces acetate production, which alters the host immune response by mitigating the suppression of anti-inflammatory IL-10(67). In addition to bacterial-specific targets, we can also enhance the host’s ability to modulate bacterial metabolism (Fig. 2). The host utilizes zinc to intoxicate GAS by inhibiting bacterial glycolytic enzymes phosphofructokinase and glyceraldehyde-3-phosphate dehydrogenase. Therapies or supplementation that enhance this natural metal-mediated defense could induce metabolic failure (74). However, systemic zinc supplementation can disrupt tightly regulated zinc homeostasis, and carries safety concerns, which require rigorous definition of therapeutic windows and delivery strategies. Other approaches, such as metal chelators, represent an expanding frontier in anti-virulence strategies (75).

Modulating the host environment or response can suppress GAS virulence by making niches less permissive (76). In severe invasive infections, host hyperinflammation driven by metabolic dysregulation is a key driver of pathology. Small-molecule inhibitors of host LDH may temper lactate overproduction and mitigate cytokine storms (Fig. 2). However, direct inhibition of host LDH carries risk because LDH is essential for systemic metabolic homeostasis. Any lactate-targeting approach would require highly tissue-specific or pathway-selective modulation.

Vaccination represents the ultimate host-directed strategy. The Combo5 vaccine (76), incorporating antigens such as arginine deiminase, aims to pre-emptively neutralize the metabolic enzymes that facilitate niche establishment.

### Challenges and future directions for therapeutic development

Translating these therapeutic strategies faces some challenges. GAS exhibits metabolic redundancy; inhibiting one pathway (e.g., LDH) may simply reroute carbon flux through another (e.g., PFL) (65). This necessitates combination therapies targeting multiple nodes. Furthermore, phenotypic heterogeneity within bacterial populations means a single treatment may not be effective against all cells in an infection, contributing to tolerance and persistence. This is particularly relevant in biofilms, where nutrient gradients create metabolically distinct subpopulations with increased drug tolerance. This heterogeneity extends to pathways beyond central carbon metabolism. For instance, *in vivo* selection favors mutants with alterations in lipid metabolism that change membrane composition and indirectly attenuate virulence (19), demonstrating how compensatory adaptations in auxiliary metabolic networks can impact treatment outcomes (19). Future work must prioritize identifying conserved essential metabolic vulnerabilities and developing multi-target approaches that prevent escape and resistance (77). The integration of systems-level models, such as genome-scale metabolic networks, can help predict these vulnerabilities and design effective combination strategies.

## CONCLUDING REMARKS

The fitness of GAS across diverse host niches is determined by its ability to sense available carbon sources and deploy appropriate metabolic pathways. This decision-making process directly commands virulence factor expression and immune evasion strategies. The research advances of the past 5 years provide clarity on the mechanistic links between metabolism and virulence. While we have made progress, fundamental gaps remain in our understanding of how metabolic decision-making translates to disease outcomes:

- What are the precise mechanisms that allow GAS to rapidly reprogram its metabolism between distinct host niches?
- How do central carbon metabolites function as direct signals to orchestrate virulence and immune evasion in real-time?
- Can the metabolic vulnerabilities exposed by these findings be effectively targeted without triggering compensatory resistance mechanisms?

Future research must prioritize several key areas to build on this foundation. Integrating genome-scale metabolic models, single-cell transcriptomics, and metabolomics will be crucial to move beyond bulk population averages to map metabolic heterogeneity *in vivo* and predict essential vulnerabilities. In concert with this work, we must also explore expanded metabolic contexts such as the host microbiome, co-infections, host metabolic diseases (e.g., diabetes), and host nutritional status, which fundamentally alter the nutrient landscape. Defining the precise mechanisms of metabolite-induced immunomodulation, particularly the *in vivo* relevance of modifications such as histone lactylation and the enzyme targets of acetate-mediated metabolic reprogramming, will, in turn, clarify how bacterial metabolism drives immunopathology. Finally, translating these insights requires developing combination therapies that target metabolic hubs alongside traditional virulence factors or antibiotics to overcome redundancy and tolerance. Understanding and targeting GAS metabolism offers a promising path to disrupting the pathogenic cycle of this formidable human pathogen.
